# High-Mobility Group Box-1 Induces Decreased Brain-Derived Neurotrophic Factor-Mediated Neuroprotection in the Diabetic Retina

**DOI:** 10.1155/2013/863036

**Published:** 2013-05-20

**Authors:** Ahmed M. Abu El-Asrar, Mohd Imtiaz Nawaz, Mohammad Mairaj Siddiquei, Abdullah S. Al-Kharashi, Dustan Kangave, Ghulam Mohammad

**Affiliations:** Department of Ophthalmology, College of Medicine, King Saud University, King Abdulaziz University Hospital, Old Airport Road, P.O. Box 245, Riyadh 11411, Saudi Arabia

## Abstract

To test the hypothesis that brain-derived neurotrophic factor-(BDNF-) mediated neuroprotection is reduced by high-mobility group box-1 (HMGB1) in diabetic retina, paired vitreous and serum samples from 46 proliferative diabetic retinopathy and 34 nondiabetic patients were assayed for BDNF, HMGB1, soluble receptor for advanced glycation end products (sRAGE), soluble intercellular adhesion molecule-1 (sICAM-1), monocyte chemoattractant protein-1 (MCP-1), and TBARS. We also examined retinas of diabetic and HMGB1 intravitreally injected rats. The effect of the HMGB1 inhibitor glycyrrhizin on diabetes-induced changes in retinal BDNF expressions was studied. Western blot, ELISA, and TBARS assays were used. BDNF was not detected in vitreous samples. BDNF levels were significantly lower in serum samples from diabetic patients compared with nondiabetics, whereas HMGB1, sRAGE, sICAM-1, and TBARS levels were significantly higher in diabetic serum samples. MCP-1 levels did not differ significantly. There was significant inverse correlation between serum levels of BDNF and HMGB1. Diabetes and intravitreal administration of HMGB1 induced significant upregulation of the expression of HMGB1, TBARS, and cleaved caspase-3, whereas the expression of BDNF and synaptophysin was significantly downregulated in rat retinas. Glycyrrhizin significantly attenuated diabetes-induced downregulation of BDNF. Our results suggest that HMGB1-induced downregulation of BDNF might be involved in pathogenesis of diabetic retinal neurodegeneration.

## 1. Introduction

 Diabetic retinopathy, a vision-threatening disease, is classically regarded as microvasculopathy. However, recent evidence suggests that diabetic retinopathy is a progressive neurodegenerative disease in which visual dysfunction is initiated early after the onset of diabetes and progresses independently of the vascular lesions [1–4]. However, the molecular mechanisms underlying the diabetes-induced retinal neurodegeneration and dysfunction are still not well understood. Recent studies revealed that diabetic retinal neurodegeneration is associated with oxidative stress resulting from excess generation of reactive oxygen species as well as inflammation [3, 5].

 Brain-derived neurotrophic factor (BDNF), a protein belonging to the neurotrophin family, is expressed in retinal ganglion cells and Müller cells [6] and is important for the survival of retinal ganglion cells [7]. BDNF is important in neural development and cell survival and is essential to molecular mechanisms of synaptic activity [8]. Recent studies suggested that the early retinal neuropathy of diabetes involves the reduced expression of BDNF and can be ameliorated by an exogenous supply of this neurotrophin [1, 3]. It was also demonstrated that the reduction of BDNF in the diabetic retina was attenuated by the antioxidant lutein, indicating that this change was partly caused by excessive oxidative stress [3].

 High-mobility group box-1 (HMGB1) is a nonhistone DNA-binding nuclear protein that is highly conserved during evolution. It stabilizes nucleosome formation and facilitates gene transcription. Necrotic cell death can result in passive leakage of HMGB1 from the cell as the protein is then no longer bound to DNA. In addition, HMGB1 can be actively secreted by different cell types, including activated monocytes and macrophages, mature dendritic cells, natural killer cells, and endothelial cells. Extracellular HMGB1 functions as a proinflammatory cytokine [9–12]. Released HMGB1 signals through the receptor for advanced glycation end products (RAGE), a member of the immunoglobulin superfamily of receptors, leading to activation of the transcription factor nuclear factor kappa B (NF-*κ*B), which may alter gene transcription and lead to the upregulation of proinflammatory cytokines, chemokines, and adhesion molecules and intensifies cellular oxidative stress [9–13], processes that may play a role in the pathogenesis of diabetic retinal neurodegeneration and dysfunction. Therefore, recently, RAGE has been implicated in the pathogenesis of various diabetic complications, via oxidative stress [13, 14]. The causal relationship between persistent neuroinflammation and neurodegenerative process is becoming increasingly recognized [15]. Strong evidence indicates that chronic, low-grade inflammation is implicated in the pathogenesis of diabetic retinopathy [16, 17]. It was also demonstrated that HMGB1 provides the link between chronic neuroinflammation and progressive neurodegeneration in neurodegenerative diseases, such as Parkinson's disease [15]. In addition, it was reported that extracellularly released HMGB1 protein mediates postischemic damage of the brain and retina and that inhibiting or knockdown of HMGB1 attenuated postischemic neurodegeneration [18–21].

 In previous studies, we demonstrated that HMGB1 was upregulated in the vitreous fluid and epiretinal membranes from patients with proliferative diabetic retinopathy (PDR) as well as in the retinas of diabetic mice. In addition, we demonstrated significant positive correlations between levels of HMGB1 and levels of inflammatory biomarkers such as monocyte chemoattractant protein-1 (MCP-1) and soluble intercellular adhesion molecule-1 (sICAM-1) in vitreous fluid from patients with PDR [22–24]. Glycyrrhizin (GA), an ingredient of the licorice roots, has long been known to exhibit glucocorticoid-like anti-inflammatory actions by inhibiting 11*β*-hydroxysteroid dehydrogenase. More recently, GA has also been shown to bind to and inhibit cytokine-like activities of HMGB1 [25].

 In this study, we explored the hypothesis that BDNF-mediated neuroprotection is reduced by HMGB1 in the diabetic retina. To test this hypothesis, we measured the levels of BDNF, HMGB1, soluble RAGE (sRAGE), biomarkers of inflammation and endothelial dysfunction including MCP-1, and sICAM-1 and the oxidative stress and lipid peroxidation marker thiobarbituric acid reactive substances (TBARS) in the vitreous fluid and serum from a series of patients with PDR. In addition, we investigated the expression of BDNF, HMGB1, the synaptic vesicles protein synaptophysin, TBARS, and the apoptosis executer enzyme cleaved caspase-3 in the retinas of diabetic rats. We also examined the effect of intravitreal administration of HMGB1 on the retinas of normal rats and whether constant GA intake suppresses diabetes-induced changes in BDNF expression.

## 2. Materials and Methods

### 2.1. Vitreous and Paired Serum Samples Collection and Preparation

Undiluted vitreous fluid samples (0.3–0.6 mL) and paired serum samples were obtained from 46 patients with PDR during pars plana vitrectomy. The indications for vitrectomy were traction retinal detachment and/or nonclearing vitreous hemorrhage. The diabetic patients were 35 males and 11 females, whose ages ranged from 22 to 80 years with a mean of 53.9 ± 12.8 years. The duration of diabetes ranged from 7 to 32 years with a mean of 16.4 ± 5.6 years. Twenty-four patients had insulin-dependent diabetes mellitus, and 22 patients had noninsulin-dependent diabetes mellitus. At presentation, the fasting blood glucose was uncontrolled in 15 patients and controlled in 40 patients. Twenty-six patients were receiving treatment for hypertension. The control group consisted of 34 patients who had undergone vitrectomy for the treatment of rhegmatogenous retinal detachment (RD) with no proliferative vitreoretinopathy. Controls were free from systemic disease and were 23 males and 11 females whose ages ranged from 12 to 82 years with a mean of 47.8 ± 16.8 years. Vitreous samples were collected undiluted by manual suction into a syringe through the aspiration line of vitrectomy, before opening the infusion line. The samples were centrifuged (5000 rpm for 10 min, 4°C) and the supernatants were aliquoted and frozen at −80°C until assay. Blood was collected after an overnight fast, and serum was obtained by centrifugation and stored at −70°C. The study was conducted according to the tenets of the Declaration of Helsinki, and informed consent was obtained from all patients. The study was approved by the Research Centre, College of Medicine, King Saud University.

### 2.2. Animals

All procedures with animals were performed in accordance with the ARVO statement for use of animals in ophthalmic and vision research and were approved by the institutional animal care and use committee of the College of Pharmacy, King Saud University. Adult male Sprague Dawley rats, 8-9 weeks of age weighting in the range of 210–230 g, were overnight fasted and streptozotocin (STZ; 65 mg/kg in 10 mM sodium citrate buffer, pH 4.5; Sigma, St. Louis, MO, USA) was injected intraperitoneally. Equal volumes of citrate buffer were injected in nondiabetic animals. Measuring of blood glucose concentrations and body weight was started after 3 days of STZ injection. Diabetes was confirmed by assaying glucose concentration in the blood taken from tail vein. Rats with glucose levels >250 mg/dL were considered to have diabetes. After 4 weeks of diabetes, animals were anesthetized by intraperitoneal injection of an overdose of chloral hydrate and sacrificed by decapitation. Retinas were dissected out, snap frozen, and stored at −80°C until use. Similarly, retinas were obtained from age-matched nondiabetic control rats.

### 2.3. Intravitreal Injection of HMGB1

Sprague Dawley rats (220–230 g) were kept under deep anesthesia, and sterilized solution of recombinant HMGB1 (5 ng/5 *μ*L; R&D Systems, Minneapolis, MN, USA) was injected into the vitreous of the right eye. For the control, the left eye received 5 *μ*L of sterile phosphate buffer saline (PBS). The animals were sacrificed 4 days after intravitreal administration, and the retinas were carefully dissected, snap frozen in liquid nitrogen, and stored at −80°C until analyzed.

### 2.4. Glycyrrhizin Treatment

Sprague Dawley rats were made diabetic as previously described. Diabetic rats were divided into 2 groups: the rats in group I received normal drinking water without any supplementation, and group II received drinking water supplemented with glycyrrhizic acid (150 mg/kg/day, Santa Cruz Biotechnology, Inc., Santa Cruz, CA, USA) immediately after establishment of diabetes. Each group consisted of 8–12 rats. After 4 weeks of diabetes, the rats were sacrificed, the eyes were removed, and retinas were isolated and frozen immediately in liquid nitrogen and stored at −80°C until analyzed.

### 2.5. Enzyme-Linked Immunosorbent Assay (ELISA) Kits

 ELISA kits for human BDNF (Quantikine Brain-Derived Neurotrophic factors Factor, Cat. no. DBD00), human MCP-1 (Quantikine Human Monocytes Chemotactic Protein-1, Cat. no. DCP00), human sRAGE (Quantikine Human Receptor for Advance glycation End products, Cat. no. DRG00) and human sICAM-1 (Quantikine Human Soluble Intercellular Adhesion Molecules-1, Cat. no. DCD540) were purchased from R&D Systems. An ELISA kit for HMGB1 (human high-mobility group box-1, Cat. no. ST51011) was purchased from IBL International GMBH (Hamburg, Germany). The detection limits for BDNF, MCP-1, sRAGE, sICAM, and HMGB1 were 20, 5, 4.12, 96, and 200 picograms/mL (pg/mL), respectively. The ELISA plate readings were done using FLUOstar Omega-Microplate reader from BMG Labtech, Offenburg, Germany. 

### 2.6. Oxidative Stress Marker Assay Kit

The assay kit for the oxidative stress and lipid peroxidation marker TBARS (Cat. no. 10009055) was purchased from Cayman Chemical Company, Ann Arbor, MI, USA. 

### 2.7. Measurement of BDNF, MCP-1, sRAGE, sICAM, and HMGB1 in Human Vitreous and Serum and BDNF in Rat Retinas

The quantifications of the level of BDNF, MCP-1, sRAGE, sICAM, and HMGB1 in the vitreous and serum and in the rat retinas were determined using specific ELISA kits according to the manufacturer's instruction. For each ELISA kit, the undiluted standard served as the highest concentration and calibrator diluents served as the blank. Depending upon the detection range of the ELISA kit and the expression level of the particular molecule, vitreous and serum samples were either directly used or diluted with calibrator diluent supplied with ELISA kit. 

For measurement of BDNF in the vitreous, 50 *μ*L of undiluted samples was added to ELISA plates for analysis. For serum, samples were diluted 25-fold, 5-fold, 2-fold, and 20-fold for BDNF, MCP-1, sRAGE, and sICAM-1 measurements, respectively. 100 *μ*L, 200 *μ*L, 50 *μ*L, and 100 *μ*L of diluted sample for BDNF, MCP-1, sRAGE and sICAM were added into each of the ELISA plates for the analysis. For measurement of BDNF in rat retinas, 200 *μ*g of rat retinal homogenate was used and added into each of the ELISA plates for the analysis. For the quantification of HMGB1 within the high sensitivity range, 50 *μ*L of diluents buffer (Dilbuf, IBL International) was added to each well of the plate followed by the addition of 50 *μ*L of 2-fold diluted sample. Following sample incubation into the wells of ELISA plates, secondary antibodies against BDNF, MCP-1, sRAGE, sICAM and HMGB1 conjugated to horseradish peroxidase were added to each well of the ELISA plate. After incubation, substrate mix solution was added for color development. The reaction was stopped by the addition of 2 N sulfuric acid and optical density (OD) was read at 450 nm in microplate reader. Each assay was performed in duplicate. Using the 4-parameter fit logistic (4-PL) curve equation, the actual concentration for each sample was calculated. For the samples that have been diluted, the correction read from the standard curve obtained using 4-PL was multiplied by the dilution factors to get the actual reading for each sample.

### 2.8. Measurement of TBARS in Human Serum and Rat Retinas

The steps for the measurement of TBARS in serum and in rat retinal homogenate were followed as per the manufacturer instructions that use the principle of formation of adduct between malondialdehyde (MDA) and thiobarbituric acid (TBA) under high temperature (95°C) and acidic condition and color developed is measured colorimetrically. Color reagent to be used was prepared by mixing TBA with TBA acetic acid and TBA sodium hydroxide (supplied with the kit). In a 10 mL tube, 100 *μ*L undiluted serum and retinal homogenate were mixed with 100 *μ*L of sodium dodecyl sulphate (SDS). The color reagent (4 mL) was added in each respective tube and was boiled for 1 hour. Tubes were cooled immediately on ice for 10 minutes and were centrifuged for 10 minutes at 1600 ×g at 4°C. The upper clear solution was loaded on 96-well clear plates and the color was measured at 530 nm using FLUOstar Omega-Microplate reader (BMG Labtech). 

### 2.9. Western Blot Analysis

Retinas were homogenized in a Western lysis buffer (30 mM Tris-HCL; pH 7.5, 5 mM EDTA, 1% Triton X-100, 250 mM sucrose, 1 mM sodium vanadate, and protease inhibitor cocktail). The protease inhibitor used was “Complete without EDTA” (Roche, Mannheim, Germany). The lysate was centrifuged at 14,000 ×g for 10 min at 4°C, and the supernatant was collected. Protein content was assayed by DC protein assay (Bio-Rad Laboratories, Hercules, CA, USA). The tissue lysateS containing 50 *μ*g protein were separated on 10–15% SDS-polyacrylamide gels and were transferred onto polyvinylidene difluoride (PVDF) membranes. The blots were blocked with TBST (20 mM Tris-HCl; pH 7.6, 136 mM NaCl, and 0.1% Tween-20) containing 5% nonfat milk. 

For detection of BDNF, synaptophysin, cleaved caspase-3, and HMGB1, the membrane was incubated overnight at 4°C with BDNF mouse monoclonal anti-BDNF (1 : 500, Cat no. SC-65513, Santa Cruz), goat polyclonal antisynaptophysin (1 *μ*g/mL, Cat. no. AF-5555, R&D Systems), rabbit monoclonal anticleaved caspase-3 (1 : 300, Cat. no. MAB835, R&D Systems) and rabbit polyclonal anti-HMGB1 (1 : 1000, Cat. no. ab18256, Abcam). After overnight incubation with primary antibodies, the membranes were washed four times with TBS-T (5 min each). For BDNF, the membrane was incubated at room temperature for 1.5 h with anti-mouse secondary horseradish peroxidase-conjugated antibody (1 : 2000, SC-2005, Santa Cruz), for synaptophysin with anti-goat secondary horseradish peroxidase-conjugated antibody (1 : 2000, SC-2768, Santa Cruz), and for cleaved caspase-3 and HMGB1 with anti-rabbit secondary horseradish peroxidase-conjugated antibody (1 : 2000, SC-2004, Santa Cruz). After incubations with secondary antibodies, membranes were washed four times with TBS-T (5 min each) and the immunoreactivity of bands was visualized on a high-performance chemiluminescence machine (G: Box Chemi-XX8 from Syngene, Synoptic Ltd. Cambridge, UK) by using enhanced chemiluminescence plus Luminol (sc-2048, Santa Cruz) and quantified by densitometric analysis using image processing and analysis in GeneTools (Syngene by Synoptic Ltd. Cambridge, UK). For loading control, membranes were stripped and incubated with a mouse monoclonal anti-*β*-actin antibody (1 : 2000, SC-2048, Santa Cruz) and all the remaining steps were followed as detailed above. All data from the three independent experiments were expressed as a ratio to mean intensity. 

### 2.10. Statistical Analysis

 The nonparametric Mann-Whitney *U* test was used to compare means from two independent study groups. Pearson correlation coefficients were computed to investigate correlations between continuous variables. A *P* value less than 0.05 indicated statistical significance. SPSS version 15.0 program was used for the statistical analysis.

## 3. Results

### 3.1. Levels of BDNF, HMGB1, sRAGE, sICAM-1, MCP-1, and TBARS in Patients with PDR and Nondiabetic Control Subjects

BDNF was not detected in vitreous samples from patients with PDR and nondiabetic control patients. BDNF, HMGB1, sRAGE, sICAM-1, MCP-1, and TBARS were detected in all serum samples from patients with PDR and nondiabetic controls. Mean levels of BDNF in serum samples from patients with PDR were significantly lower than those in nondiabetic control patients (*P* = 0.015). On the other hand, mean levels of HMGB1, sRAGE, sICAM-1, and TBARS were significantly higher in serum samples from patients with PDR than those in nondiabetic controls (*P* < 0.001; *P* = 0.008; *P* = 0.019; *P* = 0.011, resp.). Mean levels of MCP-1 did not differ significantly between patients with PDR and nondiabetic control patients (*P* = 0.836) (Table 1).

### 3.2. Correlations

**Figure 1:**
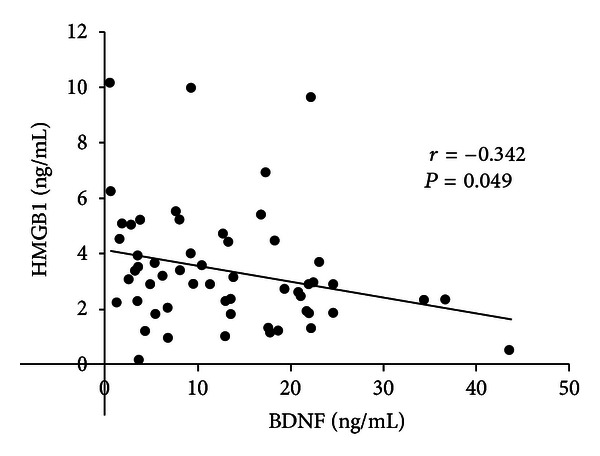
Correlation between serum levels of high-mobility group box-1 (HMGB1) and brain-derived neurotrophic factor (BDNF). There is a significant inverse correlation between serum levels of HMGB1 and BDNF.

 There was a significant inverse correlation between serum levels of BDNF and HMGB1 (*r* = −0.324; *P* = 0.049) (Figure 1). There were significant positive correlations between serum levels of TBARS and sRAGE (*r* = 0.335; *P* = 0.018), sICAM-1 (*r* = 0.303; *P* = 0.032), and MCP-1 (*r* = 0.344; *P* = 0.012). There was a significant positive correlation between serum levels of sRAGE and sICAM-1 (*r* = 0.431; *P* < 0.001) (Table 2).

### 3.3. Relationships between Serum BDNF, HMGB1, sRAGE, sICAM-1, MCP-1, and TBARS Levels and Clinical Variables at Presentation in Patients with PDR

 We examined the correlations between serum BDNF, HMGB1, sRAGE, sICAM-1, MCP-1, and TBARS levels and clinical parameters at presentation including fasting blood sugar, triglycerides, total cholesterol, HDL-cholesterol, LDL-cholesterol, and creatinine. There were significant positive correlations between serum levels of MCP-1, and triglycerides (*r* = 0.402; *P* = 0.0231) and total cholesterol (*r* = 0.519; *P* = 0.003). No other significant correlations were detected.

 There were no significant relationships between serum levels of BDNF, HMGB1, sRAGE, sICAM-1, MCP-1 and TBARS and type of diabetes treatment (Table 3). The mean serum levels of sRAGE in patients receiving antihypertensive agents were significantly higher than those in other patients (Table 4). 

### 3.4. Severity of Hyperglycemia in Rats

 The body weights of the rats with diabetes were lower and their blood glucose levels were more than fourfold higher compared with age-matched normal control rats (180 ± 22 versus 250 ± 28 g and 453 ± 32 versus 111 ± 12 mg/dL, resp.). Treatment of the diabetic rats with GA for one month did not change these metabolic variables in diabetic rats (167 ± 25 versus 178 ± 22 g and 449 ± 36 versus 475 ± 32 mg/dL, resp.). 

### 3.5. Effect of Hyperglycemia on the Expression of BDNF, HMGB1, TBARS, Synaptophysin, and Cleaved Caspase-3 in Rat Retinas

**Figure 2:**
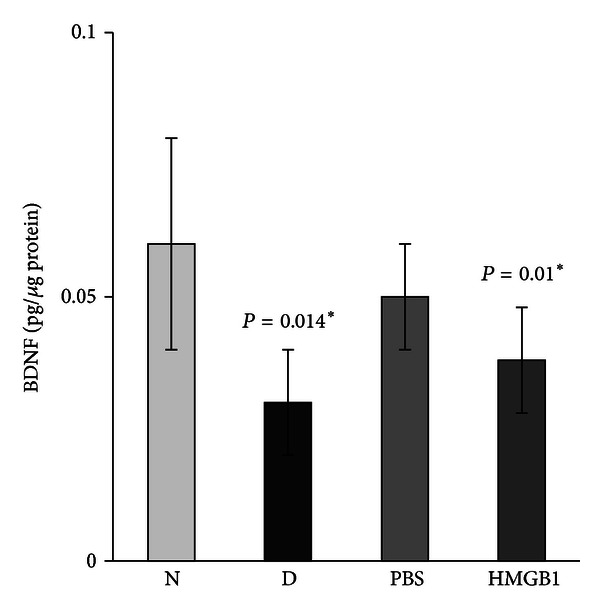
Enzyme-linked immunosorbent assay for brain-derived neurotrophic factor (BDNF) in rat retinas. There is a significant decrease in the expression of BDNF in the retinas of diabetic rats (D) compared with the nondiabetic control rats (N). Intravitreal administration of high-mobility group box-1 (HMGB1) induced a significant downregulation of the expression of BDNF compared with intravitreal administration of phosphate buffer saline (PBS). Each experiment was repeated 2 to 3 times with fresh samples (*n* = 6).

**Figure 3:**
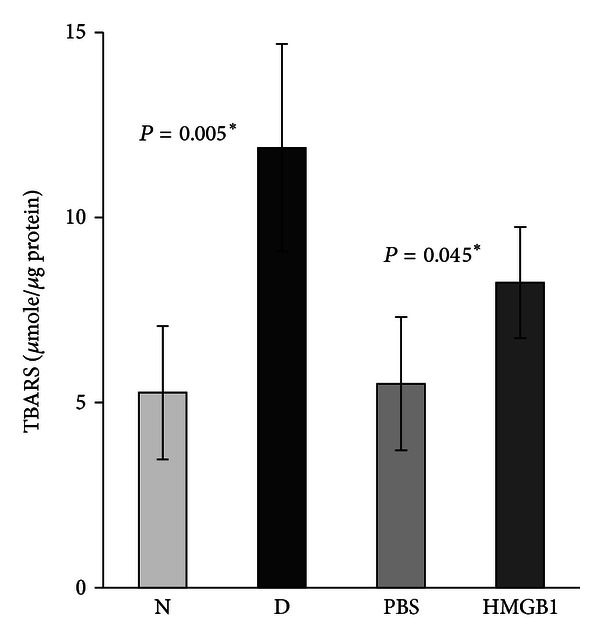
Levels of thiobarbituric acid reactive substances (TBARS) are significantly higher in the retinas of diabetic rats (D) compared with the nondiabetic control rats (N). Intravitreal administration of high-mobility group box-1 (HMGB1) induced a significant upregulation of the levels of TBARS compared with intravitreal administration of phosphate buffer saline (PBS). Each experiment was repeated 2 to 3 times with fresh samples (*n* = 6).

**Figure 4:**
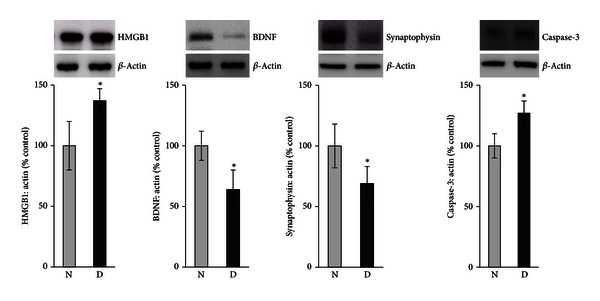
Western blot analysis of high-mobility group box-1 (HMGB1), brain-derived neurotrophic factor (BDNF), synaptophysin, and cleaved caspase-3 in rat retinas. There is a significant increase in the expression of HMGB1 and cleaved caspase-3 and a significant decrease in the expression of BDNF and synaptophysin in the retinas of diabetic rats (D) compared with the nondiabetic control rats (N). Each experiment was repeated 2 to 3 times with fresh samples (*n* = 6).

 Quantification of BDNF levels in nondiabetic controls and the retinas of rats with diabetes was done with the use of ELISA. BDNF protein levels in the retinas of animals with diabetes (0.03 ± 0.01 pg/*μ*g protein) were significantly lower than those in nondiabetic controls (0.06 ± 0.02 pg/*μ*g protein) (*P* = 0.014; Mann-Whitney *U* test) (Figure 2). The generation of TBARS in diabetic retinas (11.88 ± 8.2 *μ*mole/*μ*g protein) significantly increased compared with nondiabetic controls (5.27 ± 1.8 *μ*mole/*μ*g protein) (*P* = 0.005) (Figure 3). We quantified the expression of HMGB1, BDNF, synaptophysin, and cleaved caspase-3 by Western blot analysis. Densitometric analysis of the bands revealed a significant increase in HMGB1 (*P* = 0.01) and cleaved caspase-3 (*P* = 0.004) and a significant decrease in BDNF (*P* = 0.046) and synaptophysin (*P* = 0.003) in diabetic retinas compared with nondiabetic controls (Figure 4).

### 3.6. Effect of Intravitreal Administration of HMGB1 on the Expression of BDNF, HMGB1, TBARS, Synaptophysin, and Cleaved Caspase-3 in Rat Retinas

**Figure 5:**
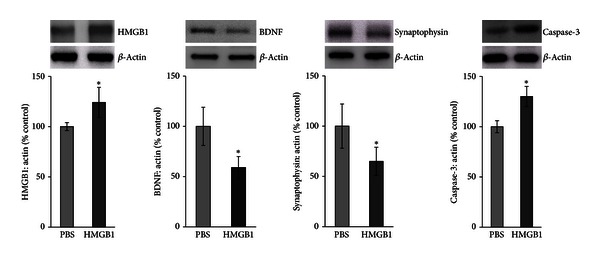
Western blot analysis of rat retinas. Intravitreal administration of high-mobility group box-1 (HMGB1) induced a significant upregulation of the expression of HMGB1 and cleaved caspase-3 and a significant downregulation of the expression of brain-derived neurotrophic factor (BDNF) and synaptophysin compared with intravitreal administration of phosphate buffer saline (PBS). Each experiment was repeated 2 to 3 times with fresh samples (*n* = 6).

 ELISA demonstrated that intravitreal administration of HMGB1 in normal rats induced significant downregulation of the expression of BDNF in the retinas (0.038 ± 0.01 pg/*μ*g protein) compared with controls (0.05 ± 0.01 pg/*μ*g protein) (*P* = 0.01) (Figure 2). HMGB1 injection induced significant upregulation of the generation of TBARS in the retinas (8.24 ± 1.5 *μ*mole/*μ*g protein) compared with controls (5.51 ± 1.9 *μ*mole/*μ*g protein) (*P* = 0.045) (Figure 3). Western blot analysis for HMGB1, BDNF, synaptophysin, and cleaved caspase-3 showed that intravitreal administration of HMGB1 in normal rats significantly increased the expression of HMGB1 (*P* = 0.001) and cleaved caspase-3 (*P* = 0.004) and significantly decreased the expression of BDNF (*P* = 0.001) and synaptophysin (*P* = 0.001) compared with controls (Figure 5). 

### 3.7. HMGB1 Inhibitor Glycyrrhizin Attenuates the Effect of Diabetes

**Figure 6:**
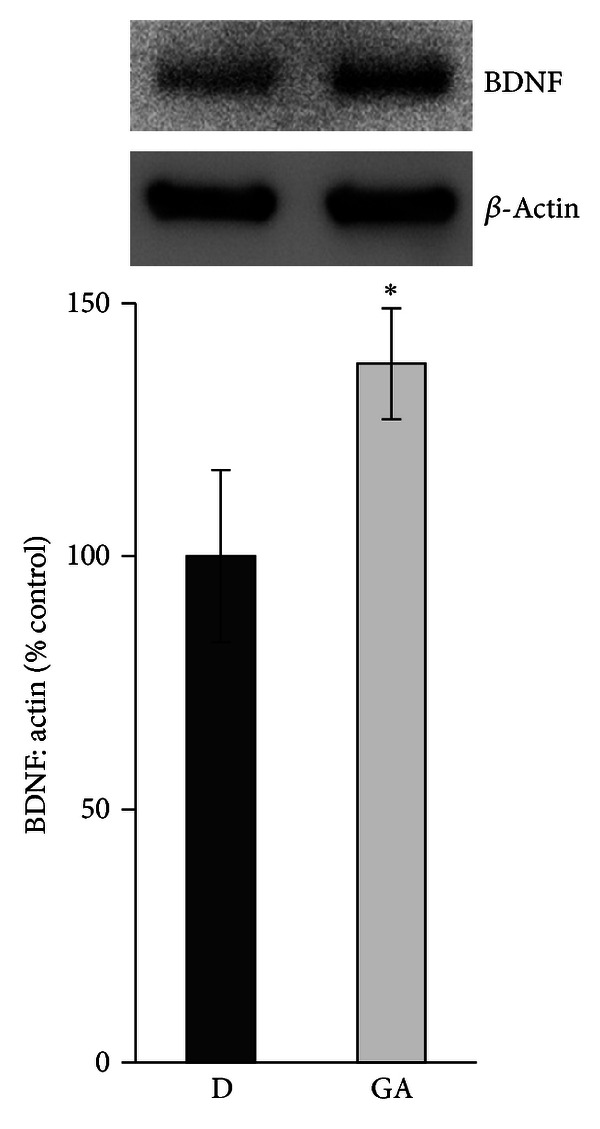
Diabetes-induced downregulation of brain-derived neurotrophic factor (BDNF) is prevented by constant intake of glycyrrhizic acid (GA) as shown by Western blot analysis. Each experiment was repeated 2 to 3 times with fresh samples (*n* = 6).

Western blot analysis was used to assess the effect of GA on diabetes-induced alterations of BDNF in the retinas of rats. Constant GA intake from the onset of diabetes significantly attenuated diabetes-induced downregulation of BDNF (*P* = 0.048) (Figure 6). In a previous study, we demonstrated that constant GA intake from the onset of diabetes significantly attenuated diabetes-induced upregulation of HMGB1 in the retinas of rats [26].

## 4. Discussion

 In the present study, we investigated the correlations between the levels of BDNF and the levels of HMGB1 in the vitreous fluid and serum from patients with PDR and in the retinas of rats with diabetes. We also investigated the effect of intravitreal administration of HMGB1 on the retinas of rats. We demonstrated that BDNF levels were below the detection limit of our test system in the vitreous fluid. BDNF was significantly downregulated in the serum from patients with PDR and in the retinas of rats with diabetes, whereas HMGB1 was significantly upregulated. We also found a significant inverse correlation between the levels of BDNF and HMGB1 in the serum. Intravitreal administration of HMGB1 to normal rats induced a significant downregulation of BDNF. Our results are consistent with previous reports that demonstrated reduced levels of the protein and mRNA of BDNF in streptozotocin-induced diabetic rat retinas [1, 3]. Sasaki et al. [3] reported that excessive oxidative stress is responsible for the reduced BDNF levels in the retinas from rats with diabetes. The antioxidant lutein prevented reactive oxygen species generation, visual impairment, BDNF depletion, and neuronal cell apoptosis in the diabetic retina. Furthermore, intraocular administration of BDNF rescued retinal dopaminergic amacrine cells from neurodegeneration in rats with diabetes [1]. The findings suggest that the early retinal neuropathy of diabetes involves the reduced expression of BDNF, whose deficiency is associated with a number of neurodegenerative disorders [27]. We showed that the protein level of BDNF was also reduced in the retinas by intravitreal injection of HMGB1, whose levels are elevated in the vitreous fluid and epiretinal membranes from patients with PDR as well as in the retinas of diabetic animals [22–24]. In addition, the HMGB1 inhibitor GA attenuated diabetes-induced upregulation of HMGB1 and downregulation of BDNF in the retinas of rats. Our findings suggest that diabetes-induced decrease of BDNF in the retina seems to be mediated by HMGB1.

 BDNF, in addition to its role in neuronal health, plays a systemic role in glucose metabolism. In animals, BDNF is involved in insulin resistance, reduces food intake, and lowers blood glucose levels in obese diabetic mice [28]. It was also demonstrated that, compared with thiazolidinediones, BDNF potently ameliorates pancreatic dysfunction, fatty liver, and energy expenditure, thereby exerting favourable antidiabetic effects in type 2 diabetic mice [29]. It was also reported that high levels of glucose, but not insulin, inhibit the output of BDNF from the human brain [30]. These findings suggest that BDNF may have a potential as a unique hypoglycemic agent for the treatment of diabetes. In line with earlier reports [30, 31], serum levels of BDNF in patients with PDR were significantly reduced compared to control nondiabetic patients. In contrast to BDNF, we demonstrated elevated levels of HMGB1 in the serum from patients with PDR. Our results are consistent with previous reports that demonstrated elevated levels of HMGB1 in the serum from patients with type 1 and type 2 diabetes [32, 33] and that higher serum HMGB1 levels were associated with greater prevalence and severity of albuminuria. In addition, HMGB1 levels were positively associated with markers of low-grade inflammation and endothelial dysfunction [33].

 Synaptophysin is an integral membrane protein of the synaptic vesicles. It possibly serves multiple functions in synaptic vesicle formation and exocytosis, playing an important role in neurotransmitter delivery. It is widely used as one of the synaptic function markers and is also thought to be closely related to synaptogenesis and synaptic plasticity during neural tissue development. Synaptophysin knockout mice exhibited a significant decrease in synaptic vesicles in retinal rod photoreceptors, which disturbs neurotransmitter release and synaptic network activity [34]. Previous studies demonstrated that 1 month of diabetes decreases the retinal expression of synaptophysin [3, 35, 36] and that the antioxidant lutein prevented synaptophysin reduction and avoided increase in cleaved caspase-3 in the diabetic retina [3] suggesting that local oxidative stress has a neurodegenerative influence in diabetic retina. In the present study, we demonstrated that, similar to diabetes, HMGB1 caused a significant decrease in the synaptic vesicle protein synaptophysin and a significant increase in the activated cleaved caspase-3 in the retina of normal rats.

 Activation of HMGB1/RAGE signaling axis is important in promoting proinflammatory pathways considered to play an important role in diabetes-induced retinal neuroinflammation [9–12, 16, 17]. Interaction of HMGB1 with RAGE results in activation of NF-*κ*B, release of cytokines and chemokines, expression of adhesion molecules, and induction of oxidative stress [9–14]. In our laboratory, we recently demonstrated that diabetes induced significant upregulation of the expression of HMGB1, RAGE, activated NF-*κ*B, and intercellular adhesion molecule-1 (ICAM-1) in the retinas of rats and that intravitreal administration of HMGB1 in normal rats mimics the effect of diabetes. In addition, coimmunoprecipitation studies showed that diabetes increases the interaction between HMGB1 and RAGE [26]. These findings suggest a pathogenic role of HMGB1 in the development of diabetic retinopathy through RAGE and activation of NF-*κ*B. Recently, it was reported that chronic neuroinflammation may be a driving force of progressive neurodegeneration and that HMGB1 provides the link between chronic neuroinflammation and progressive neurodegeneration in neurodegenerative diseases, such as Parkinson's disease [15].

 In the present study, we report that sRAGE and sICAM-1 levels were significantly upregulated in the serum from patients with PDR and that MCP-1 levels did not differ between patients with PDR and nondiabetic control subjects. Our results are consistent with previous reports that demonstrated increased circulating levels of sRAGE [32, 37, 38] and sICAM-1 [32, 39–41] in both patients with type 1 and type 2 diabetes mellitus compared to nondiabetic controls. Pham et al. [41] demonstrated increased serum concentration of sICAM-1 in patients with type 2 diabetes mellitus compared with healthy subjects, whereas serum concentrations of the chemokine MCP-1 were not increased. It has been suggested that human sRAGE production might be derived from alternative RNA splicing as well as by release from the full-length RAGE receptor by proteinases [42]. It was also demonstrated that the engagement of HMGB1 with RAGE promotes the shedding of the receptor and that high levels of soluble forms of RAGE correlate with high levels of chronic ongoing inflammation [42]. Furthermore, circulating sRAGE levels may reflect enhanced tissue RAGE expression in diabetic vasculature [43]. In the present study, we identified a significant positive correlation between the serum levels of sRAGE and the biomarker of endothelial activation and dysfunction sICAM-1 consistent with a previous study [32]. These findings suggest that RAGE signaling pathway is involved in the pathogenesis of endothelial activation and dysfunction in diabetes and that these factors may be coregulated in diabetic retinopathy.

 sRAGE, a truncated form of the receptor, binds ligands with affinity equal to that of cellular RAGE. It, therefore, has the ability to prevent RAGE signaling acting as a “decoy” by binding ligands and preventing them from reaching the cell surface RAGE. *In vitro*, sRAGE added to cultured cells blocked the effects of RAGE ligands on expression of inflammatory markers, cellular migration and proliferation, and cytotoxicity [44]. sRAGE has successfully been used in a variety of animal disease models to antagonize RAGE-mediated pathologic processes [44, 45]. Several studies showed that sRAGE might beneficially impact early vascular and neuronal dysfunction in the diabetic retina. Systemic administration of sRAGE inhibits blood-retinal barrier breakdown, leukostasis, and expression of ICAM-1 in the retina of diabetic animals [46]. In addition, attenuation of the RAGE axis with sRAGE ameliorated retinal neuronal dysfunction and reduced the development of capillary lesions in a murine model of nonproliferative diabetic retinopathy [47]. Our results suggest that elevated levels of sRAGE in the serum from patients with PDR potentially negatively regulate inflammation and that sRAGE is secreted extracellularly as a negative feedback mechanism to limit diabetes-induced retinal vascular and neuronal dysfunction.

Lipid peroxidation is considered a hallmark of oxidative stress. In our study, there was a significant increase in lipid peroxidation in the serum from patients with PDR and in the retinas of diabetic rats as measured by TBARS formation. In addition, intravitreal administration of HMGB1 to normal rats induced significant upregulation of TBARS in the retina. These results are in agreement with several studies that reported elevated levels of circulating TBARS in diabetic subjects [48–50] and that the expression of TBARS is increased in the retinas of diabetic rats [51]. Our analysis showed significant positive correlations between the serum levels of TBARS and sRAGE, sICAM-1, and MCP-1. Our results are consistent with a previous report that demonstrated a significant positive correlation between plasma levels of TBARS and sICAM-1 in patients with type 1 diabetes mellitus [52] and that hyperglycemia-induced oxidative stress induces an increase of circulating ICAM-1 levels [53] and the expression of RAGE in human endothelial cells [54]. On the basis of our findings, we propose a causal relationship linking hyperglycemia, activation of HMGB1/RAGE signaling axis, neuroinflammation, oxidative stress, and diabetic retinal neurodegeneration. 

 In conclusion, these data suggest that diabetes-induced increased oxidative stress, downregulation of BDNF and synaptophysin, and upregulation of cleaved caspase-3 were also induced by HMGB1. The HMGB1 inhibitor GA attenuated diabetes-induced downregulation of BDNF in the retinas of rats. Collectively, our present data suggest that blocking HMGB1 signaling pathways might be a novel therapeutic strategy for neuronal dysfunction in vision-threatening diabetic retinopathy.

## Figures and Tables

**Table 1:** Comparisons of mean levels for brain-derived neurotrophic factor (BDNF), high-mobility group box-1 (HMGB1), monocyte chemoattractant protein-1 (MCP-1), soluble intercellular adhesion molecule-1 (sICAM-1), soluble receptor for advanced glycation end products (sRAGE), and thiobarbituric acid reactive substance (TBARS) in serum samples.

Disease group	BDNF (ng/mL) (mean ± s.d)	HMGB1 (ng/mL) (mean ± s.d)	MCP-1 (pg/mL) (mean ± s.d)	sICAM (ng/mL) (mean ± s.d)	sRAGE (pg/mL) (mean ± s.d)	TBARS (*μ*M) (mean ± s.d)
PDR	10.2 ± 7.7	4.4 ± 2.1	267.34 ± 174.0	205.2 ± 99.1	898.1 ± 536.9	29.0 ± 20.7
RD	16.7 ± 10.3	2.2 ± 1.4	267.29 ± 187.7	157.0 ± 45.7	589.7 ± 320.9	17.2 ± 6.1
Mean comparison *P*-value	0.015*	<0.001*	0.836	0.019*	0.008*	0.011*

*Statistically significant at 5% level of significance.

PDR: proliferative diabetic retinopathy; RD: rhegmatogenous retinal detachment.

**Table 2:** Pearson correlation coefficients between serum levels of the studied factors.

	BNDF	HMGB1	MCP-1	TBARS	sICAM-1
HMGB1	*r* = −0.324				
	*P* = 0.049*				
MCP-1	*r* = 0.089	−0.156			
	*P* = 0.519	0.250			
TBARS	*r* = −0.115	0.121	0.344		
	*P* = 0.413	0.382	0.012*		
sICAM	*r* = −0.103	−0.016	0.168	0.303	
	*P* = 0.450	0.907	0.219	0.032*	
sRAGE	*r* = −0.253	0.252	0.241	0.335	0.431
	*P* = 0.070	0.068	0.085	0.018*	<0.001*

*Statistically significant at 5% level of significance.

Where the row and column meet is the correlation coefficient and the *P* value for the two variables.

BDNF: brain-derived neurotrophic factor; HMGB1: high-mobility group box-1; MCP-1: monocyte chemoattractant protein-1; TBARS: thiobarbituric acid reactive substance; sICAM-1: soluble intercellular adhesion molecule-1; sRAGE: soluble receptor for advanced glycation end products.

**Table 3:** Relationship between serum levels of the studied factors and type of diabetes treatment in patients with proliferative diabetic retinopathy.

	Use of insulin		*P* value
	Yes (mean ± s.d)	No (mean ± s.d)	
BDNF (ng/mL)	10.1 ± 8.4	10.3 ± 7.2	0.749
HMGB1 (ng/mL)	4.7 ± 2.4	4.2 ± 1.9	0.546
MCP-1 (pg/mL)	271.8 ± 198.7	263.4 ± 155.2	0.835
TBARS (*μ*M)	31.6 ± 25.0	26.9 ± 16.9	0.842
sICAM-1 (ng/mL)	196.0 ± 83.9	214.5 ± 113.4	0.673
sRAGE (pg/mL)	1013.6 ± 565.3	787.9 ± 496.1	0.206

*Statistically significant at 5% level of significance.

BDNF: brain-derived neurotrophic factor; HMGB1: high-mobility group box-1; MCP-1: monocyte chemoattractant protein-1; TBARS: thiobarbituric acid reactive substance; sICAM-1: soluble intercellular adhesion molecule-1; sRAGE: soluble receptor for advanced glycation end products.

**Table 4:** Relationship between the serum levels of the studied factors and presence or absence of hypertension in patients with proliferative diabetic retinopathy.

	Presence of hypertension		*P* value
	Yes (mean ± s.d)	No (mean ± s.d)	
BDNF (ng/mL)	10.1 ± 7.7	10.3 ± 8.0	0.907
HMGB1 (ng/mL)	4.4 ± 2.3	4.5 ± 1.8	0.565
MCP-1 (pg/mL)	244.9 ± 156.0	300.2 ± 199.4	0.489
TBARS (*μ*M)	24.2 ± 11.2	35.7 ± 28.5	0.356
sICAM-1 (ng/mL)	213.0 ± 118.9	194.1 ± 61.8	0.756
sRAGE (pg/mL)	1050.5 ± 536.2	686.5 ± 474.2	0.017*

*Statistically significant at 5% level of significance.

BDNF: brain-derived neurotrophic factor; HMGB1: high-mobility group box-1; MCP-1: monocyte chemoattractant protein-1; TBARS: thiobarbituric acid reactive substance; sICAM-1: soluble intercellular adhesion molecule-1; sRAGE: soluble receptor for advanced glycation end products.
